# Supplementary material for: Are carbon emissions trading and green financial instruments synergistic? -Comprehensive quantitative research based on content analysis
Source: PLoS One. 2024 Mar 7;19(3):e0298601. doi: 10.1371/journal.pone.0298601 (PMC10919601; doi:10.1371/journal.pone.0298601)
Supplement: S1 Data — (ZIP) [file pone.0298601.s001.zip › Data/地级市Z_F工作报告.zip!/╣π╢1⁄2/╔ε█┌╩╨/└┤╫╘╣1⁄2╓┌║┼í╛┬φ┐╦╩2╛▌═°í┐_2007.docx]

各位代表：

　　现在，我代表深圳市人民政府向大会作政府工作报告，请予审议，并请各位政协委员和其他列席人员提出意见。

　　一、2006年经济社会发展基本情况

　　2006年，是实施“十一五”规划的第一年，全市人民在党中央、国务院、省委、省政府和市委的正确领导下，以邓小平理论和“三个代表”重要思想为指导，认真落实科学发展观，国民经济和社会发展取得新的成就，实现了“十一五”的良好开局。

　　——国民经济健康平稳增长，四大支柱产业主导作用更加突出。全年本市生产总值达5684亿元，增长15%；规模以上工业增加值2723亿元，增长17.2%；全社会固定资产投资1272亿元，增长7.7%；社会消费品零售总额1671亿元，增长16.2%；进出口总额2374亿美元，增长29.9%，其中出口总额1361亿美元，增长34.1%。四大支柱产业增加值占GDP的比重达54.4%，提高4.6个百分点。

　　——固定资产投资适度增长，消费拉动和创新驱动作用不断增强。固定资产投资增幅低于GDP增幅7.3个百分点；社会消费品零售总额增幅近10年来首次高于GDP增幅。实现高新技术产品产值6294亿元，其中具有自主知识产权的占58.9%。全社会研发投入占GDP的比例达3.4%。专利申请量增长42%，达到29728件，居全国第二；其中发明专利申请量增长75.3%，申请量居全国第一，PCT国际专利申请量稳居全国榜首。我市被评为全国“品牌之都”，已有58个产品获得“中国名牌”称号，21件商标获得“中国驰名商标”称号，华为和中兴的程控交换机被评为中国世界名牌产品。

　　——整体经济效益稳步提升，资源能源消耗水平持续下降。效益深圳统计正式纳入统计体系，开始成为衡量经济增长模式转变的重要尺度。2006年效益深圳综合指数稳步提高。每平方公里土地产出GDP为2.91亿元，提高0.38亿元。全社会劳动生产率提高7.5%。全市万元GDP能耗、水耗以及全年化学需氧量排放量、二氧化硫排放量分别有不同程度下降。地方财政一般预算收入500.88亿元，增长21.5%，上划中央收入首次突破千亿，达1180亿元。

　　——社会更加文明和谐,人民群众更多地享受到经济发展的成果。各项社会事业取得新进步，城市生活环境质量进一步提升，名列“2006中国城市生活质量排行榜”第一位。城市登记失业率控制在2.31%的较低水平。人均GDP达到8619美元，居全国大中城市第一。居民人均可支配收入增长5%。社会治安形势明显好转，全市刑事案件下降10.2%,八类暴力性案件下降29.9%。信访量、群体性事件明显下降。安全事故总起数下降33.1%，死亡人数下降8.3%,直接经济损失下降25.3%。成功申办2011年第26届世界大学生运动会，成为历史上最年轻的主办城市，这是我们深圳的光荣，是全市人民的光荣，也是我们伟大祖国的光荣！

　　这些成就，标志着和谐深圳效益深圳的建设加快推进，标志着我市朝着国际化城市和国家创新型城市的目标，又迈出了坚实的一步！

　　2006年，我们按照市委四届四次全会的总体部署，突出重点，明确责任，强化落实，政府工作报告提出的“1348”工作目标及116项重点工作全面完成。

　　一是进一步强化重大发展战略的制定和实施，全力推进自主创新和循环经济。

　　把自主创新作为城市发展的主导战略。认真贯彻市委关于建设国家创新型城市的1号文件和《深圳经济特区循环经济促进条例》，出台了相关配套政策、规划和近期实施方案。召开全市进一步加快自主创新全面推进循环经济大会，全市上下形成了共同关注、全面推进自主创新和循环经济的良好态势。

　　创建国家创新型城市工作取得积极进展。深化科技管理体制改革，建立了政府决策、专家评审、中介审核的政府科技研发资金配置新机制。设立实施“非共识”技术创新项目资助计划等13类科技计划，科技研发资金链覆盖到技术创新链的各个环节。设立产业发展和创新人才奖。中科院深圳先进技术研究院正式成立，大学城国家级重点实验室和深圳留学生基地建设启动，市科技图书馆建成试运行，公共技术平台和科技信息服务平台进一步完善。实施知识产权战略、品牌战略和标准化战略取得明显成效，通过“国家知识产权试点城市”验收。第八届高交会国际化、专业化、市场化水平进一步提高。

　　大力发展循环经济。各区、各部门在产业发展、城市建设、社会管理方面认真践行循环经济理念，启动了一批循环经济试点项目，积极探索以贴息等方式扶持节能产品和项目的发展。开展“鹏城减废”行动，推广清洁生产，推进建筑节能改造和“四节一环保”住宅小区建设，成为全国首个住宅产业化试点城市。探索建设“能效电厂”，加快推进重点用能单位节能降耗。大力实施节约用水条例，严格用水管理，建成一批中水利用示范工程，特区内用水连续两年负增长。节能、节水、绿色消费正在转化为政府、企业、社会的自觉行动。

　　二是产业结构进一步优化，城市整体功能得到提升。

　　在集成电路、平板显示、化合物半导体等高新技术产业领域引进一批高端项目，产业链不断向上延伸。哈飞深圳研发生产基地的新车型正式下线，比亚迪汽车研发制造基地即将在深圳推出中高档新型轿车。机电一体化产业取得新的发展，国家生物产业基地、软件出口基地、坝光精细化工园区的规划建设有序推进。高档家具、服装、黄金珠宝等优势传统产业在国内国际市场的竞争力明显提升，产业集聚基地建设步伐加快。支持金融业的政策措施取得成效，金融业出现近十年来最强劲的增长。金融中心区、金融产业园区和香港金融业后台服务基地建设稳步推进，11家金融机构落户深圳。股权分置改革基本完成，上市公司质量明显提高。“全国保险创新发展试验区”建设取得积极成果。市商业银行和深国投引进战略投资者工作顺利完成。深圳港集装箱吞吐量1847万标准箱，增长14%，继续稳居世界第四；深圳机场旅客吞吐量1836万人次，增长12.7%，连续五年位居国内第四。盐田港保税物流园区正式运作，六大物流园区建设提速，西部港区进行了资源整合和功能调整，机场国际货运业务取得突破，物流业的发展环境进一步改善。第二届文博会在规模、水平和质量上有了重大提升，品牌效应初步形成。怡景动漫基地、深圳文化创意产业园、大芬油画村等已成为我市文化产业发展的重要集聚地。国际旅游城市建设步伐加快，东部华侨城等旅游重大项目顺利推进，全年接待过夜境外游客713万人次，国际旅游收入22.65亿美元，分别居国内城市第一和第四位。成功举办世界牙科联盟2006年会，是我市历史上所承办的国际化水平最高的会议。

　　114个枢纽型、功能型、公益性重大基础设施建设进展顺利。京广深港客运专线、厦深铁路、龙华客运中心站前期工作取得较大进展。轨道交通5条线的规划、报批和建设工作加快推进。港口、航道、机场飞行区扩建、口岸建设和改造、疏港道路等基础设施项目建设进展顺利。南坪快速一期、盐排高速等一批现代交通路网骨干项目相继建成通车，深港西部通道深圳湾公路大桥全线贯通。深南大道改造工程、福龙路、南光路以及部分市政道路配套设施改造项目建设顺利展开。

　　资源能源保障能力进一步增强。东江水源二期、北线引水、铁岗水库扩容等一批水源工程进展顺利。LNG项目一期工程建成投产，城市天然气利用工程全面铺开。积极争取省网支持，基本保障了城市用电需要。加快推进电网和电厂建设，全年投资超过40亿元，东部电厂一期、老虎坑垃圾焚烧发电厂投入运营。与中海油、中广核公司的战略合作取得实质性进展。启动城市能源规划和战略研究，积极建立成品油储备的长效机制和应急体系，国家成品油战略储备基地规划建设加快。

　　三是进一步深化改革、扩大开放，经济社会发展活力不断增强。

　　大力推进改革攻坚，30项改革任务得到全面落实。以提供更多更好的公共产品和公共服务为目标，以分类改革为突破口，全面启动事业单位改革，基本完成124家事业单位转企，以及党政机关事业单位所办270家企业的划转工作。建立了相对集中有效的行政审批机制，重大项目从立项到开工的审批时限由原来的316－386个工作日缩短到110个工作日。启动第四轮行政审批制度改革。积极推进国有企业内部经营机制转换和劳动、人事、分配制度改革，国有企业各项经济效益指标位居全国前列。完善促进民营经济发展的体制机制，出台了加快民营经济发展的若干措施，民营经济发展出现了加快和加强的新趋势。第三轮市区财政体制改革顺利实施，逐步理顺市、区政府的事权和财权，为建立现代公共财政体制奠定了基础。加快人口管理体制改革，市、区、街道、社区四级人口管理网络体系建设全面推进，居住证和出租屋管理立法工作取得积极进展，人口管理工作进一步加强。

　　外贸出口结构进一步优化，一般贸易、高新技术产品和机电产品出口所占比重稳步上升。外商投资企业增资活跃，已有141家世界500强企业投资深圳。积极发展服务外包产业，成为首批“中国服务外包基地城市”。大力实施“走出去”战略。“深企闯天下”取得明显成效。对外直接投资协议金额增长接近五成，对外承包工程完成营业额增长50%以上。积极开展贸易投资推介活动，国际经贸科技合作取得新的成效。建立完善反倾销与产业损害预警系统，为企业提供对外贸易信息和维权服务。

　　积极推进区域经济合作，深港两地金融合作进一步深入，“深港创新圈”建设提上议程，深港双方在城市规划、跨境基础设施建设、口岸通关、食品安全、环境保护、文化教育、公务员培训，以及旅游和投资环境推介等领域的合作更加深入。深澳合作实现历史性突破，建立了政府间沟通协调机制。全方位拓展国内市场，积极参与泛珠三角地区在基础设施、能源、经贸、旅游、劳务等方面的合作。认真做好对口扶持，有序引导产业转移。大力发展海铁联运、江海联运，经济腹地进一步扩大。

　　四是不断改善社会民生，强力推进人民群众最关心、最直接、最现实利益问题的解决。

　　制订并实施新三年就业再就业政策体系，加强公益性岗位开发力度，实现了“零就业家庭”出现一户解决一户。大幅提高最低工资标准，继续完善社会保障体系，劳务工医疗保险参保人数达到343万，工伤保险基本实现全覆盖。初步形成多部门联动打击恶意欠薪的长效机制，切实保障劳动者权益。我市被评为全国“最受农民工欢迎的十大城市”。积极构建企业履行社会责任的制度框架。完善住房保障制度，加强经济适用房、公共租赁住房建设和管理，建立全面覆盖“双困”家庭的廉租住房制度。坚决贯彻落实国家加强房地产市场调控的有关政策，规范房地产市场交易秩序,保持房地产市场健康、理性发展。推进供水中间层接收工作，明显减轻了小区居民的负担。

　　全面推出义务教育均衡发展的政策措施，促进教育公平，提升教育整体质量和水平。落实人口管理“1＋5”文件，使所有符合条件的孩子平等享受义务教育的权利。加快高中学校建设，新增5300多个高中学位。加强医疗服务价格管理，实施医疗分配制度改革，全年平均门诊人次费用和平均住院人次费用分别下降10%。推进公共卫生重大项目建设，全面加强社区卫生服务，全年新增医疗机构1046家，社康中心覆盖率达到75.5%，“看病难、看病贵”问题有所缓解。大力加强流动人口计生服务，流动人口政策生育率提高15.7个百分点，圆满完成省下达的人口计生各项任务。

　　启动公安系统公务员专业化改革，创新派出所勤务管理模式，一线和基层警力得到加强。积极推进科技强警，加强重点人口和出租屋动态管控，提升社会治安防控效能。食品安全“五大工程”和“四大体系”建设稳步推进，在全省食品安全综合评价中，我市名列第一。积极开展非正常上访集中整治行动，推行领导干部带案下访、开门接访，妥善解决了一批重大疑难信访问题，全市信访秩序明显好转。

　　广泛开展社会主义荣辱观教育实践活动。未成年人和大学生思想教育工作不断加强，“关爱行动”、“支援灾区”、“军民共建”等群众性精神文明创建活动蓬勃开展，丛飞先进事迹在全国产生强烈反响，市民综合素质、社会道德风尚和城市文明水平稳步提升。文化事业和文化产业健康发展，公共文化服务体系不断完善，大剧院、图书馆新馆、中心书城三大重点文化设施投入使用。成功举办“2006深圳国际钢琴协奏曲比赛”等一批高水平的文化活动，“文博会”、“读书月”、“市民文化大讲堂”等成为响亮的城市文化品牌。鼓励公益性文化活动进社区、工厂和校园，推动公益性文化设施免费开放服务。

　　五是全面推开基层基础和城市管理“双年活动”，不断提高城市现代化、国际化水平。

　　市、区两级投入近20亿元，用于基层基础建设。全市新增社区办公和服务设施14万平方米，无偿移交社区居委会用房约2万平方米，基层工作条件明显改善。积极创新社区管理服务模式，加快推行“居站分设”、“一站多居”和“居企分开”，初步理顺了基层管理体制机制，社区居民自治和服务功能得到强化。

　　城市管理工作扎实推进，一批商业旺区环境综合整治进展顺利，人民南商圈改造基本完成，主要街区旧房“穿衣戴帽”工作全面展开，城中村和社区环境质量明显改善。户外广告专项整治顺利推进。数字化城管系统特区内平台建成试运行，被确定为全国首批数字化城管试点城市。顺利通过国家环境保护模范城市复查和国家卫生城市复查，在全国首个被授予“国家生态园林示范城市”。水环境治理取得积极进展，城市生活污水处理率提高11个百分点，生活污水处理能力、处理率和工业废水排放达标率均列全省第一，初步实现大沙河、福田河、新洲河、凤塘河、盐田河、荔枝湖等“五河一湖”不黑不臭，深圳河水质明显好转，布吉河水质有所改善。LNG清洁能源转换工作顺利推进，燃煤电厂脱硫、汽车尾气和垃圾填埋场空气污染治理加强，获得“保护臭氧层示范城市”称号。严厉打击环境违法行为，强制淘汰落后生产工艺。环保审查否决不符合环保和产业发展要求的投资项目2388宗，关闭污染企业435家。

　　大力实施公交优先，特区内公交的服务质量明显提高，被评为“全国实施公交优先战略示范城市”。福田交通枢纽中心完成主体工程，新建7个公交总站，扩展完善公交线网，公共中小巴全部平稳退出特区。实现过境疏港货运交通“东进东出，西进西出”的重大转变。稳步推进城市现有道路交通体系改造，推行交通事故快理快赔制度，在机动车增加17.3万辆的情况下，全市道路通行状况没有恶化。

　　深圳2030城市发展策略经市人大常委会审议通过，成为指导我市城市规划建设的法定依据。城市总体规划修编工作全面启动，城市近期建设规划及2006年度实施计划正式实施，特区外八大组团分区规划和全市工业布局规划、深圳湾15公里海滨休闲带等一批重要专项规划顺利完成，光明新城、龙华新城、体育新城、东部新城及大鹏半岛等重点片区规划设计加快进行。积极探索建立城市规划督察巡视制度，利用卫星遥感技术对城市规划实施情况进行动态监测，强化了对基本生态控制线的管理和保护。实施最严格的土地管理，召开了首次全市土地管理工作会议，加强政府对土地资源的统筹和调控,实现了城市土地的统一管理。加大查违、清无工作力度，拆除违法建筑159万平方米，违法建筑增量得到遏制；21万多家无证无照非法经营户中，超过99%得到有效清理整治。全面完成宝安、龙岗两区城市化转地，城中村改造工作稳步推进。

　　六是更加注重政府自身建设，着力提高行政执行力。

　　全面推行政府目标管理，探索开展政府绩效评估工作，切实加强责任追究。建立行政监督联席会议制度和行政违法违规问题移送制度，完善行政监督链条，对重大投资项目审批实施专门的电子监督，行政监察工作明显加强。落实重大事故和重大事件行政机关责任检讨制，试行部门责任“白皮书”制度。全面加快电子政务建设，大力推进国家电子政务综合试点城市各项工作。行政服务大厅工作效率和服务水平不断提高。

　　进一步完善公共行政管理决策机制，颁布实施市政府重大决策公示暂行办法、市政府常务会议工作规则、行政听证办法等规章制度，规范政府重大事项的决策程序，发挥政府法律顾问作用，防范法律风险。积极推进政府职能调整和行政资源整合，合理划分政府部门和市、区两级政府事权，实施街道综合执法试点，推动了城市管理重心逐步有序下移。创新社会组织管理体制，加强了民间组织管理力量，为进一步推进政府职能转变创造了有利条件。

　　加强民主法制建设，推进依法行政。认真执行市人大及其常委会的决议并报告工作，顺利办结市人大重点建议5件，人大代表建议541件，政协提案620件。自觉接受人大的法律监督、工作监督，接受人民政协、民主党派的民主监督和新闻媒体的舆论监督。对于涉及城市长远发展和市民重大利益的事项，认真听取各方意见和建议，严格履行听证、公示程序。完善行政许可实施配套办法，实现了全市行政许可实施法定化。通过依法行政和法制教育，机关事业单位、企业和市民的责任意识逐步加强，被评为全国“四五”普法先进城市。

　　外事、侨务、港澳台务、民族宗教、档案、双拥、气象、民防、残疾人等各项事业都取得新进步。

　　各位代表，回顾过去一年的工作，我们深感成绩来之不易。在此，我代表深圳市人民政府，向奋斗在各条战线的全市广大干部群众和外地来深建设者，向各位人大代表、政协委员、各民主党派、工商联和社会各界人士，向中央和各省区市驻深单位，驻深解放军、武警官兵，向所有关心支持深圳建设的港澳同胞、台湾同胞、海外侨胞和国际友人，表示崇高的敬意和衷心的感谢！

　　我们也清醒地看到，我市经济社会发展还存在着一些亟待解决的困难和问题，突出表现在：一是经济社会发展条件的约束日益趋紧，解决能源、资源等瓶颈制约的力度有待进一步加大；二是安全生产形势依然严峻，一些单位安全意识不够强，重大安全事故时有发生；三是人民群众关心的一些热点难点问题还没有得到很好解决，“看病难、看病贵”、房价较快上涨等还没有得到根本解决；四是社会治安和维稳综治问题还很突出，利益协调、矛盾纠纷排查和危机处置需要更加主动、有效；五是政府执行力建设的一些问题还没有真正解决，公共行政管理和公共服务水平有待进一步提高。对这些问题，我们将认真对待，决不回避，采取有效措施，切实加以解决，绝不辜负人民的期望和重托！

　　二、2007年的主要目标任务

　　按照党的十六届六中全会、中央经济工作会议和省委九届十次全会精神及市委四届五次全会的总体部署，今年我们要进一步增强责任感、紧迫感和危机感，更加突出重点、统筹兼顾，关注民生、促进和谐，加快创新、营造环境，优化产业、增强后劲，推动转型、提高效率，廉洁从政、狠抓落实，加快推进经济增长方式转变，加强城市人文精神建设，努力开创科学发展、和谐发展的新局面。

　　综合考虑各方面的因素，今年我市经济社会发展的主要预期目标和约束指标是：本市生产总值增长13%；万元GDP建设用地、能耗和水耗分别下降10%、2.75%和4%；二氧化硫和化学需氧量排放量分别削减5%和1.5%；城镇登记失业率控制在3%以内；居民人均可支配收入增长7%。

　　今年，必须重点做好以下工作：

　　（一）以高端制造业和高端服务业为龙头，以自主创新和循环经济为抓手，强力推动产业结构优化升级

　　产业结构优化升级是国民经济又好又快发展的基础，是城市综合竞争力不断提升的关键。深圳的发展奇迹就在于顺应经济规律，迅速实现了从低向高持续不断的产业优化升级，成功地跨越了不同的发展阶段。在新的起点上，我们要审时度势、抢抓机遇，积极应对企业所得税法实施带来的挑战，牢牢把握结构调整这条主线，大力发展高端制造业和高端服务业，实现新的跨越。

　　一是要加快推进高新技术产业向产业链的高端延伸，着力引进建设芯片、TFT等核心重大项目，实现通信、数字视听、软件、新型储能材料等电子信息产业从模仿加工到研发制造的转变。二是要积极发展精细化工、汽车及汽车电子、机电一体化等装备制造业。三是要加快发展生物医药、化合物半导体等战略创新产业，培育发展适合深圳特点的海洋经济和海洋产业。四是要发挥高新区的辐射带动作用，做大做强高新技术产业带，加快优势传统产业聚集基地建设，新建一批特色工业园区。逐步引导企业将劳动密集的加工环节向外转移，把总部、研发中心、采购中心及财务中心留在深圳，实现新的发展。五是大力发展循环经济，进一步提高产业投资项目的土地、市场、节能、环保等准入门槛，坚决淘汰高污染、高消耗的低端产业；新上项目必须进行资源能源消耗审核和环境影响评价，不符合节能环保标准的，坚决不上。加快电子、能源、建筑等重点行业技术标准和技术规范的修订调整，建立健全资源节约的标准体系，推进循环经济示范小区建设，创建“国家生态示范园区”，使循环经济型、资源节约型和环境友好型产业成为我市产业结构中的靓丽风景线。

　　大力提升源头创新能力。进一步加快科技管理体制改革，充分发挥中科院深圳先进技术研究院的创新功能，加快重点实验室建设，提升高等院校教学和科研水平，完善各类科技公共平台，全面提升大学、科研机构、企业与政府四位一体的研究开发机制在科技创新中的基础性作用。全力推进“深港创新圈”建设，完善深港科技合作机制。大力实施知识产权战略、品牌战略和标准战略，争创“国家知识产权示范城市”。以承办“中国国际人才交流会”为契机，加强人才高地和引智工作政策体系建设，优化人才环境，不断提高我市对各类高端人才的吸引力。进一步提高“高交会”办展水平和实效，充分发挥其创新资源交流重要平台的作用。

　　加快发展现代金融、现代物流、服务外包、创意设计、品牌会展、高端旅游等八大高端服务业。抓住金融业扩大开放的机遇，积极推动金融创新，争取国家有关部门支持，创建银行业、证券业创新发展试验区。加快金融聚集区和金融产业服务基地项目建设，大力培育金融控股集团，吸引更多金融机构总部和后台处理中心落户深圳。支持促进多层次资本市场的建立和完善，加大创新型中小企业上市工作力度，推进中小企业上市路线图计划。鼓励金融机构完善股权激励机制，完成上市公司股权分置改革和清欠工作。促进银企沟通，创新融资工具，大力发展债券市场和金融租赁、金融信托业务。鼓励保险业开发新险种，拓展新业务，加快建设“全国保险创新发展试验区”。完善征信系统，优化金融生态环境，保障金融安全。

　　大力推进海港、空港建设。理顺部门职责，为港口、船公司及各类物流企业提供更加优惠的政策和高效服务；完善疏港交通条件，降低物流成本，完善珠江水系驳船集装箱运输网络，构建稳定的港口喂给网络，不断提升深圳港作为国际集装箱枢纽港的辐射力、竞争力。积极拓展机场客运服务市场，大力发展航空货运业务，巩固提高深圳机场区域性航空货运中心地位。加快盐田港保税物流园区和六大物流园区建设，继续做好空港保税物流园区申报和建设，着力引进一批高附加值的重点物流项目，探索保税区向自由贸易园区转型。

　　编制完成文化产业发展战略规划，加快文化基础设施规划建设步伐，办好第三届“文博会”、促进文化产业跨越式发展。大力发展现代商贸业，着力优化商业网点布局，提升商贸业服务水平。积极推进具有国际水准的重点旅游项目和旅游设施建设，努力营造国际化旅游环境。完善总部经济服务体系，促进和培育本土企业做大做强，鼓励和支持国内外大企业集团在深设立总部或区域总部。

　　（二）以解决人民群众最关心、最直接、最现实的利益问题为着力点，全面推进和谐社会建设

　　要高度重视和谐社会建设，加大解决民生问题的人力、物力、财力和精力投入，尽最大努力消除社会不稳定因素，最大限度地增加社会和谐因素。

　　完善劳动力市场管理，健全公共就业服务体系，积极为农转居、失业人员以及外来工提供免费就业服务和技能培训。完善困难群体就业再就业援助体系，建立“零就业家庭”归零工作的长效机制，全面解决户籍人口大学生就业问题。继续关爱外来工，加大维权力度。实施企业工资支付情况的即时监察、欠薪预警和“黑名单”制度，完善行政司法联动机制，与珠三角各城市联手打击恶意逃薪行为，积极推进企业社会责任制度建设，构建和谐的劳动关系。

进一步完善社会保障体系。健全农民工养老保险制度，积极推进全市工伤、医疗保险全覆盖，加强社保基金管理。发展残疾人事业、老龄事业和社会慈善事业，完善社会救助机制，全面构建社会救助体系，加大对困难群体的帮扶力度。继续开展“同富裕”工程。加强房地产市场调控和监管，保障房地产的有效供给，坚决打击恶意炒楼、囤积土地房屋等行为,保持房地产市场健康发展。完善住房公积金制度，加强住房保障，着力解决低收入家庭的住房困难。

　　深入推进义务教育均衡发展，增强教育的普惠性和公平性，完善特区外基础教育规划布局。加大投入，全面提高学前教育质量和水平。加快寄宿制高中建设。规范发展民办教育，积极推进职业教育。大力推进高等教育跨越式发展，正式启动南方科技大学筹建工作，积极推进深圳大学改扩建工程，支持深职院参与国家示范性高等职业院校建设计划，加快信息学院新校区建设，着力优化大学城办学模式。加快发展继续教育，努力构建终身教育体系。

　　切实加快卫生系统重点项目、社区健康服务中心和劳务工医疗网点建设，完善市级医院在各区的布局。要尽快实现每个区至少有一所市级医院，让全市人民都能就近方便地享受高水平的医疗服务。积极推进医疗卫生体制、医疗保障制度、药品流通体制改革，完善公共卫生和社区卫生服务体系，规范医疗收费，净化医疗市场。完善社康中心管理体制机制，提高社康中心医疗服务水平。进一步加强艾滋病等重大传染病防治工作。贯彻落实中央关于全面加强人口和计划生育工作统筹解决人口问题的决定精神，加强领导，强化责任，加大投入，完善服务，构建具有深圳特色的人口计划生育工作新机制。

　　加大交通综合整治力度，下大力气解决重点区域交通拥堵严重的问题，提高交通组织管理的科学化和现代化水平，确保中心城区道路基本畅通。优化公交线网结构，提高公交覆盖率，实现公共交通便利化。加快特区外公交资源整合重组，推进公交区域专营，改变特区外公交落后的状况，逐步实现特区内外公交一体化。适当降低公交票价，减轻市民出行负担。去年，梅林关出现严重交通拥堵问题，有关部门公开向市民道歉；今后凡因政府部门工作失误或失职造成公共利益受到重大损失的，都要公开道歉，勇于承担责任，并启动监察督查程序，这是一个责任政府的基本体现！

　　要进一步吸取血的教训，狠抓安全生产责任制的落实，切实消除各类安全隐患，真正做到警钟长鸣，长抓不懈，永不言胜，用一万的努力防止万一的发生。要加大安全生产基础设施投入，完善安全规章制度，加大宣传教育力度，全面增强市民的安全意识，构筑坚实的安全防线。全力推进食品安全“五大工程”和“四大体系”建设，整顿和规范食品药品市场秩序，不断提高食品药品安全水平。

　　加强社会治安综合治理，严厉打击各类刑事犯罪。继续推进科技强警，加快电子防控工程等公安基础设施建设。强化流动人口和出租屋管理，整合社区内维稳资源，营造群防群治网络，构建社会治安立体防控体系。继续推进公安体制改革、警务运行机制创新和公安系统公务员专业化改革。进一步完善适应社会转型的利益协调、诉求表达、矛盾调处和权益保障机制。加快信访立法，创新信访工作方式方法，探索信访与行政复议联动机制，建立全市信访信息系统，引导群众通过法律途径解决争议和纠纷。加大应急物资储备和管理力度，推进应急体制机制建设，提高城市防灾、国防动员和处置突发事件的能力。

　　进一步深化以社区建设为重点的“基层基础年”活动。科学划定社区范围，理顺社区管理体制，完善公共服务设施，改善社区工作人员的待遇和办公条件，建设一支宏大的社工队伍，构建多元互动的城市社区组织管理体系，打造平安和谐社区。积极推进传统社会向公民社会转型，规范发展社会组织，鼓励社会创新，探索由政府、企业、社会共同解决社会问题的新模式，促进社会和谐。

　　加快建设和谐文化，努力形成人人讲和谐、人人促和谐的局面。要牢固树立社会主义荣辱观，大力弘扬深圳精神。积极发展文化事业，完善公共文化服务体系，丰富公益文化活动，塑造更多城市文化品牌，满足市民文化需求，保障市民文化权利。规划建设科学馆新馆、现代艺术馆、城市规划展馆，加大特区外社区文体设施建设力度。继续办好“深圳读书月”等一批品牌文化节庆活动，推动京剧、粤剧等传统文化事业发展。大力发展体育事业，积极开展群众性体育活动。加快义工队伍建设，深入开展“关爱行动”和社会志愿服务活动，营造平等友爱、融洽和谐的社会氛围。

　　（三）以筹办2011年世界大学生运动会为契机，加快重大基础设施规划建设，不断增强城市辐射力和竞争力

　　筹办2011年世界大学生运动会，是我市今后几年的一项重点工作，是对深圳国际赛事组织能力和城市管理水平、城市文明程度的一次重大考验。全市上下要立即行动起来，认真做好大运会的各项筹办工作，加快完善相关工作方案，推进“大运中心”和重点体育场馆等硬件建设，建设一流场馆，营造一流环境，提供一流服务，力争办出历史上最出色大运会。

　　优化城市功能布局，不断完善城市整体功能。高水平完成城市总体规划和土地利用总体规划修编工作，完成一批重要专项规划，精心组织实施组团分区规划和城市近期建设规划，抓紧各项法定图则编制工作，着力提升组团功能和土地利用集约化水平。实施最严格的土地管理制度，加大对土地违法违规行为的查处力度。强化土地利用计划管理，严格执行国家下达的土地利用年度计划。积极保障重大产业项目、基本住房、城市基础设施和公共服务设施的用地需求。完善产业用地政策，全面实施工业用地招拍挂，提高土地的综合利用效率。做好城市化转地收尾工作，强化全市储备用地的统一管理。加强存量土地资源和城市更新研究，抓紧开展城市地下空间资源的统一规划、开发和管理。以建设海洋大市为目标，积极推进海洋规划体系建设。

　　切实加大基础设施建设力度。加快推进国家铁路“两线两站”规划建设，推动厦深铁路深圳段全面开工。继续推进盐田港区三期扩建、大铲湾一期、蛇口港区三期建设工程，完成铜鼓航道一期，建成西部公用航道，不断完善港口基础设施建设，巩固强化深圳港的国际集装箱枢纽地位。加快推进深圳机场扩建工程，积极争取国家有关部门的支持，缓解机场空域紧张的状况，进一步提升深圳机场航空货运中心地位。全力推进轨道交通二期工程建设，实现轨道交通1号线延长段和2、3、4号线的全面开工建设，尽快完成轨道交通三期建设规划和5号线及2、3号延长线工程可行性研究，启动轨道交通三期工程6、8号线的前期论证工作，确保城市轨道交通建设目标如期完成。加快推进城市路网改造和快速公交系统建设，继续推进干线路网建设，建成福龙路和深盐二通道，进一步完善高快速路网体系。完成深圳湾口岸和福田口岸建设，加快皇岗口岸改造和文锦渡口岸旅检场地改造工程，积极推进莲塘口岸的规划工作。要加快政府历史投资项目清理工作，确保投资效果。

　　加大特区外基础设施投入力度，全力加快特区外城市化进程。以提升深圳区位优势为目标，加快推进龙华新城建设；以拓展深圳产业空间、提升产业水平为目标，加快推进以大工业区、光明工业园区为重点的东部新城、光明新城建设；以生态保护和有序开发为原则，稳步推进大鹏半岛建设发展，积极推进西部滨海地区综合战略的实施。全面推动特区外中心城区以及主要旧工业区功能置换和城中村改造。加强全市各组团之间的快速公交连接，高水平规划建设特区外各类配套设施，尽快改变特区外市政配套设施严重滞后的局面。

　　（四）以“科学、严格、精细、长效”为方针，着力提高城市综合管理水平

　　继续深入开展以环境治理为重点的“城市管理年”活动，全面推进生态市建设。实施环境保护“十百千万”行动，推进治污保洁十大工程。抓好河流污染治理，全面启动生态治河工作，实施茅洲河水环境综合整治项目和深圳河湾支流水系生态化改造工程，进一步改善河流水质。大力推进特区内污水处理厂升级改造及管网完善工程，加快特区外污水处理厂、污水截排收集干管系统和污泥处理厂建设。实施蓝天工程，加快南头半岛空气污染治理，积极联合香港、珠三角各城市对阴霾天气进行研究监测，共同推进空气污染防治工作。切实保护好、管理好基本生态控制线这一城市生态保护的“铁线”，加快妥善解决线内的历史遗留问题。加大对环境违法行为的处罚力度，发现一起，查处一起，绝不姑息。推动生态风景林、森林公园、郊野公园、市政公园、社区公园和自然保护区建设。加快建设中央商务区、深圳湾海滨15公里休闲带、福田凤塘河红树林修复示范区、大鹏半岛国家地质公园，做好重点地区、重点地段的城市设计和建筑精品设计，全面推进城市主要干道两侧的旧城旧村改造，抓好重点商业街区的市容市貌整治工作，精心打造城市亮点。

　　进一步加强城市综合管理工作。着力实施常态化、标准化、规范化管理，加快建立城市管理长效机制。全面推进城市管理街道综合执法，加强执法队伍建设，完善综合执法环境。持续开展“清无”等重点专项整治行动，加大户外广告清理。坚决查处违法建筑，确保违法建筑零增量。加快处理历史遗留违法建筑，尽快完成对1999年3月5日以前违建的处理工作，全面启动1999年3月5日至2004年10月28日期间违建的处理工作。加快特区外“数字城管”工程建设，扩大网格化管理范围，实现“数字城管”全覆盖。建立城市事件快速处理的责任机制，强化执法人员的责任意识。加强环卫工作，提高城市保洁水平，努力把深圳打造成为全国最干净的城市。

　　加快数字深圳建设，努力提高城市信息化水平。积极推进社会公共服务信息化，加快整合全市信息资源，健全公共信息资源服务体系。扎实推进电子政务，完善政府对外公共服务平台。加大信息基础设施建设力度，积极开发、应用先进的信息管理技术，加快开展下一代互联网试验网建设工作。

　　加强和完善人口管理，加快实施居住证管理办法。调整人口计划体系，实施分区域人口调控计划。加快人口综合管理信息平台建设，健全人口信息采集工作。完善市、区、街道、社区四级人口综合管理架构，落实人口和出租屋分级分类管理制度。

　　（五）以加强水电油气保障为重点，努力完善城市能源资源保障体系

　　积极构建覆盖各区、互联互通、分片调蓄的稳定供水水源网络，健全城市水源安全保障体系。继续推进东江水源工程二期、北线引水工程、大鹏半岛水源工程等水源网络项目的规划建设，加快规划建设铁岗、公明、清林径、东江下矶角等“四大水缸”，积极开展对雨洪资源、地下水资源、海水资源、中水污水资源的科学利用，提高水资源供应的应急能力。实施饮用水源一级保护区封闭管理，提高水源水质保障水平。加快特区外水务资源重组。

　　积极推动岭澳核电三期工程、抽水蓄能电站的前期工作，加紧东部电厂、前湾电厂等重点电厂项目及电网重点工程建设，彻底解决主电网供电“卡脖子”问题。完善电网处置大面积停电预案和“黑启动”机制，争取增加省网供电指标，确保电网安全稳定供电。强化电力需求预测，落实分级错峰用电、均衡用电、科学用电和节约用电。

　　开展城市可持续发展的能源战略研究，积极探索建立多层次的成品油、气储备体系，完善成品油市场应急预警机制，加快第二个LNG接收站、国家成品油深圳储备基地、天然气安全储备库等项目的可行性研究和规划建设。推进与中海油的战略合作，支持有实力、有资源的石油公司进入成品油零售终端市场。加强粮食等重要物资的储备和调控管理。

　　（六）以改革开放为动力，进一步激发深圳经济社会发展的活力

　　进一步推进社会事业和社会管理体制改革。深化事业单位改革，逐步明确事业单位的设立标准和分类定级标准，创新事业单位内部管理和人事分配制度，探索法定机构的新型组织形式，逐步建立“以事定费、以费养事”的公共服务供养新模式。深化公用事业和公用设施投资与监管体制改革，完善多元化投资机制，多渠道筹集建设资金。

　　进一步加大有利于实现社会公平的制度改革。深化改革的首要目标，是促进社会和谐，让每一位市民都能分享改革发展成果，利益受到保障，生活更有尊严。继续深化公共财政制度改革，把财政资金更多地投向民生领域，更多地投向公益性事业，更多地投向困难群体。按照“保障基本、全面覆盖”的思路，调整教育、卫生、文化等公共产品的供给结构，进一步提高公共服务的质量和水平。加大转移支付力度，构建更加完善的收入分配调节体系，稳步提高低收入者的收入水平，落实好最低工资保障和城市最低生活保障制度。

　　进一步推进市场经济体制改革。继续深化国有企业内部三项制度改革，完善企业法人治理结构，加强国有资产监管，科学动态调整国有企业的战略布局，加大国有资本运作力度，做强做大一批产业集团。切实实现不同经济主体间的公平待遇和公平竞争，全面落实支持民营经济发展的各项政策，启动民营企业创新创业公共服务平台建设，不断优化创业环境，加快培育一批拥有核心技术、竞争力强的民营龙头企业。积极推进集体经济和股份合作公司规范发展。深入整顿和规范市场经济秩序，特别要在与人民群众生命健康紧密相关的食品、药品等方面，强化市场日常监管，建立完善各项监管制度，形成长效机制。积极推进社会诚信体系建设。

　　大力完善“走出去”政策和服务体系。探索建设深圳境外经济贸易合作区，整合上下游企业，搭建中小企业海外发展平台。实施本土跨国公司培育工程，加强对企业境外投资的指导协调，重点支持一批优势企业对外直接投资、开拓重点市场、开发稀缺资源和获取先进技术，鼓励以集团军方式“走出去”。加强境外重点区域办展推介工作，积极发挥深圳驻外机构和国际友好城市的作用，逐步建立我市企业境外发展的全球经贸协作网络。进一步提高高新技术产品、高附加值产品、具有自主知识产权、自主品牌的产品和服务的出口比例。完善进出口贸易预警机制，协助企业妥善处理国际贸易摩擦，维护深圳产业和经济安全。把引进外资与调整优化产业结构有机结合起来，加强高端服务业、总部经济和重点高新技术产业项目的引进工作，力争成为区域产业链上重要的制造研发基地、采购物流基地以及服务基地。

　　加强深港跨境基础设施合作，确保西部通道和福田口岸如期建成开通；深化深港在创新圈、人民币业务、高端服务业、高等教育、公务员及专门人才培养等领域的合作。加强深澳合作、大珠三角合作和泛珠三角合作，积极参与西部大开发、振兴东北老工业基地、中部崛起和环渤海经济圈建设，探索与兄弟省市在能源、资源、科技、经贸等领域的合作，最大限度地拓展发展空间。创新完善工作机制，加强劳务合作，进一步做好对口帮扶工作。

　　（七）以提高行政执行力为核心，大力加强政府自身建设。

　　深入开展“行政绩效年”活动。加快建设“政府绩效评估系统”，今年要在16个政府职能部门及全市六区政府进行试点。凡政府重大工作、重大政策、重大改革、重大投入在出台实施一段时间后，都要对落实情况和效果进行评估。改进政府部门责任白皮书制度，扩大覆盖范围，把白皮书中规定的职能职责作为绩效评估的重要内容。除了政府及各部门自身要加强绩效评估外，还要适时引入人大、政协及第三方的绩效评估，将评估与政策调校、督查问责有机结合起来，形成科学决策、强力落实的“闭环效应”。要进一步落实“责任风暴”和“治庸计划”，在健全行政责任体系的基础上，实行更加严格的目标管理、更加严格的绩效评估，更加严格的督查督办、更加严格的责任追究，建设一个对人民负责、让人民满意的政府。

　　加快构建科学合理、精简高效的政府架构。依照市政府负责政策制定和宏观决策，区政府负责组织协调和监督检查，街道负责具体执行和事务处理的总体思路，进一步理顺市、区、街道事权划分，转变政府职能，推进行政资源下移，管理重心下移，加快建立起分工明确，授权合理的无缝化纵向公共行政管理链条。借鉴先进管理模式，逐步理顺和界定不同部门间的职能分工，重点解决政府系统内部职能交叉、责任不清、事权分离和管理真空等突出问题。大力推进以部门主办责任制、联席会议制度、行政执法协调机制为重点的部门间协调沟通工作机制建设，有效解决跨层级、跨区域、跨分管领域和跨部门的问题，减少行政资源内耗。

　　着力强化政府的公共服务职能。要把政府该管的事管好，把该办的办好，把不该管、不该办的事坚决放开，将政府工作重心转到社会管理和公共服务上来。支持行业协会、中介组织在社会管理和公共服务中发挥更大作用。积极推进第四轮行政审批制度改革，进一步清理规范非行政许可审批事项，实现审批的法定化和标准化，不断完善相对集中的行政审批机制。创新政府公共服务手段，加强行政服务大厅建设，提高政府公共服务的质量和效率。积极探索“网上审批”，以现代信息技术手段促进政府服务理念和方式创新，更好地为市民、企业和社会服务。

　　积极推进法治政府和法治社会建设。公民社会是法治社会，政府与市民均有依法行事的责任，要充分发挥法律对社会生活的规范、调整和引导作用，以法律为准绳处理各种矛盾纠纷。改进政府立法工作，增强立法工作的前瞻性和计划性，完善公众参与立法的机制、程序和方法，提高立法质量。更加重视社会管理、公共服务和民计民生方面的立法，夯实构建和谐深圳的法治基础。大力加强民主法制建设，自觉坚持向市人大及其常委会报告工作制度，接受人大的法律监督和工作监督，充分发挥市政协、各民主党派和社会各界在政治协商、民主监督和参政议政中的作用，认真办理人大建议和政协提案、建议案。做好人大制定的法律法规的实施工作，提高依法行政水平。进一步深化行政执法体制改革，整合执法资源，加强执法监督，规范执法行为，改进执法作风，切实提高执法水平。加快透明政府建设步伐，完善政府公共关系工作体制机制，健全政务公开制度、新闻发布制度和重大突发事件报道快速反应机制，最大限度地保障人民群众的知情权。

　　大力加强政风建设。良好的政风是保持和提升城市竞争力最重要的软环境、软资源。风清则气正，政通则人和。我们的政府是人民的政府，政府工作不是做给领导看，不是做给上级看。政府工作没有做好，首先是愧对人民群众，首先要对人民群众作出交代。心中有了人民群众，有了对人民群众负责的意识，工作就会积极主动，就会不等、不靠、不推、不拖，就会竭尽全力、鞠躬尽瘁。要继续发扬奋发有为的精神状态，大兴敢抓敢管、改革创新、调查研究、勤俭廉洁和团结协作之风。坚持求真务实，精简文山会海，确保今年政府全市性会议和部门工作会议以及市政府各种文件比去年减少15%。加强公务员队伍建设，推进公务员专业化改革，加强公务员学习培训，试行聘任公务员制度，完善雇员管理制度，探索建立公务员激励机制。完善财政资金管理，扩大财政资金绩效审计，提高财政资金使用效益。进一步加强廉政建设和反腐败斗争，强化监督制约，规范行政权力的运行。以解决群众反映的突出问题为重点，严肃查处违反行政纪律和侵害人民群众利益的案件，坚决纠正损害群众利益的不正之风。深入开展治理商业贿赂专项工作。大力弘扬艰苦奋斗的精神，努力降低行政成本，反对铺张浪费和奢靡之风，切实做到“为民、务实、清廉”。

　　（八）认真办好十件民生实事，不断提高民生净福利水平。

　　提高民生净福利水平，关键是要把有限的政府财力人力物力资源用在刀刃上，用在每件实事上，让民生净福利看得见、摸得着，让市民得到真正的实惠。今年，市政府将着力做好十件民生实事：

　　一是新建社康中心120家，实现社区健康服务全覆盖，使市民小病不出社区，全年平均门诊人次费用和平均住院人次费用继续有所下降，有效解决“看病难、看病贵”问题。

　　二是市区共同投入，全面完成特区外96所原村办小学设备设施的配置，全面启动校舍改扩建工程，显著改善特区外义务教育条件。

　　三是完成近期规划的336座小型垃圾转运站建设任务，实现全市建成区垃圾站的全覆盖，建立方便、快捷的垃圾收运系统。

　　四是完成两家豆制品厂主体工程建设，开工建设三家大型屠宰场，农贸市场升级改造率达到50%以上，进一步提高食品安全水平。

　　五是新增政策性住房用地60万平方米，开工、在建2.5万套经济适用房和公共租赁房，年内可用于出售和租赁的不少于6000套，实施特困家庭住房保障制度，发挥政策性住房对房地产市场的调控作用。

　　六是坚决打击企业恶意欠薪行为，加强对企业与员工签订劳动合同的督促检查，全面推广劳动合同制度和集体协商制度，劳动合同签订率达到92%以上。

　　七是建立少儿医疗保险制度，参保人数达到30万人，提高少年儿童医疗保障水平。

　　八是特区内建成区基本实现500米公交全覆盖，特区外建成区500米公交覆盖率达到60%，福田交通综合枢纽换乘中心建成并投入使用。

　　九是正式启用“12345”政府公开电话系统，服务范围逐步覆盖政府公共服务的全部领域，为市民提供全方位、全天候、高效率的政府公开电话服务。

　　十是积极推进社区公园建设，完成100个社区公园建设，优化市民居住环境，努力打造“公园之城”。

　　今年政府的各项工作安排和十件民生实事，是政府对全体市民的郑重承诺，我们一定会全力以赴，确保百分之百完成，确保百分之百兑现，向全市人民交上一份满意的答卷。

　　各位代表，当前，深圳正站在新的起跑线上，建设和谐深圳效益深圳、国际化城市和国家创新型城市，任重而道远。让我们紧密团结在以胡锦涛同志为总书记的党中央周围，高举邓小平理论、“三个代表”重要思想伟大旗帜，以科学发展观、社会主义和谐社会理论统揽全局，在市委的正确领导下，团结全市人民，振奋精神，扎实工作，为把深圳打造成为经济繁荣发达、社会和谐进步、环境优美宜人、民生幸福安康的可持续发展的全球先锋城市而努力奋斗！
